# Association of Mushrooms and Algae Consumption with Mortality among Chinese Older Adults: A Prospective Cohort Study

**DOI:** 10.3390/nu14193891

**Published:** 2022-09-20

**Authors:** Jie Shen, Mengjie He, Rongxia Lv, Liyan Huang, Jiaxi Yang, You Wu, Yuxuan Gu, Shuang Rong, Min Yang, Changzheng Yuan, Ronghua Zhang

**Affiliations:** 1 School of Public Health, The Children’s Hospital, National Clinical Research Center for Child Health, Zhejiang University School of Medicine, Hangzhou 310058, China; 2 Institute of Nutrition and Food Safety, Zhejiang Provincial Center for Disease Control and Prevention, Hangzhou 310051, China; 3 Global Center for Asian Women’s Health, Yong Loo Lin School of Medicine, National University of Singapore, Singapore 117549, Singapore; 4 Bia-Echo Asia Centre for Reproductive Longevity & Equality (ACRLE), Yong Loo Lin School of Medicine, National University of Singapore, Singapore 117549, Singapore; 5 Institute for Hospital Management, School of Medicine, Tsinghua University, Beijing 100084, China; 6 Center for Gerontology Research, Department of Social Security, Nanjing Normal University, Nanjing 211102, China; 7 Department of Public Health School, Wuhan University of Science & Technology, Wuhan 430065, China; 8 Department of Nutrition, Harvard T. H. Chan School of Public Health, Boston, MA 02115, USA

**Keywords:** mushrooms, algae, all-cause mortality, Chinese older adults, prospective study

## Abstract

Mushrooms and algae are important sources of dietary bioactive compounds, but their associations with mortality remain unclear. We examined the association of mushrooms and algae consumption with subsequent risk of all-cause mortality among older adults. This study included 13,156 older adults aged 65 years and above in the Chinese Longitudinal Healthy Longevity Survey (2008–2018). Consumption of mushrooms and algae at baseline and age of 60 were assessed using a simplified food frequency questionnaire (FFQ). We used Cox proportional hazards models to estimate the hazard ratios (HRs) and 95% confidence intervals (CIs). During 74,976 person-years of follow-up, a total of 8937 death cases were documented. After adjustment for demographic, lifestyle, and other dietary factors, participants who consumed mushrooms and algae at least once per week had a lower risk of all-cause mortality than rare consumers (0–1 time per year) (HR = 0.86; 95% CI: 0.80–0.93). Compared to participants with rare intake at both age 60 and the study baseline (average age of 87), those who maintained regular consumptions over time had the lowest hazard of mortality (HR = 0.86; 95% CI: 0.76–0.98). Our findings supported the potential beneficial role of long-term consumption of mushrooms and algae in reducing all-cause mortality among older adults. Further studies are warranted to evaluate the health benefit for longevity of specific types of mushrooms and algae.

## 1. Introduction

Mushrooms and algae are important dietary sources of bioactive compounds, including dietary fiber, minerals, polysaccharides (e.g., β-glucans, chitin), vitamins, and antioxidants [1,2,3]. Because of their unique taste and suggested health benefit, consumption of mushrooms and algae has increased considerably throughout the world, with main species including Lentinula, Pleurotus, Auricularia, Agaricus, Flammulina, Porphyra, and Laminaria [4,5]. Accumulating studies have identified the potential biological properties of edible mushrooms and algae and their extracts. For example, of all food groups, mushrooms have the highest level of ergothioneine, a sulfur-containing antioxidant, which cannot be synthesized by the human body [6,7]. Other bioactive components in mushrooms and algae extracts, such as natural polysaccharides and glucans, may be protective against cancers, immune system disorders, and beta-amyloid peptide toxicity in the brain [8]. Moreover, certain species of mushrooms and algae have also been documented as having high potential in medicinal applications [9,10].

Several observational studies have shown that higher intakes of mushrooms and algae were associated with lower risks of cancer [11], hypertension [12], type 2 diabetes [13], hyperuricemia [14], and cognitive impairment [15]. A recent meta-analysis including five prospective studies reported an inverse association between mushrooms intake and total mortality [16], although inconsistent findings were observed across studies. For example, a 27-year cohort study of 15,546 older adults in the U.S. and another 13-year follow-up cohort study conducted in 451,151 adults in European countries found that mushrooms consumption was associated with a lower risk of all-cause mortality [17,18]. However, two cohort studies in Japan have yielded nonsignificant associations [19,20]. Overall, population-based evidence regarding the potential role of mushrooms and algae intake in mortality is insufficient. 

Therefore, we used data from the Chinese Longitudinal Healthy Longevity Survey (CLHLS), a nationally representative study, to examine the prospective associations of mushrooms and algae consumption with all-cause mortality.

## 2. Materials & Methods

### 2.1. Study Population

The CLHLS is an ongoing prospective cohort study on centenarians, nonagenarians and octogenarians with a comparative group of older adults. Initiated in 1998, the CLHLS sampled community-dwelling older adults from 23 out of 31 provinces throughout China. Participants were followed up with every 2–4 years thereafter, and new participants were enrolled in each wave. Face-to-face interviews were conducted with a structured questionnaire by trained health workers. More detailed descriptions of the study design can be found elsewhere [21]. The study was approved by the Biomedical Ethics Committee of Peking University, Beijing, China (IRB00001052–13074). All participants or their proxy respondents provided informed consent before data collection. 

In the current study, we defined the 2008 cycle (*n* = 16,954) as the study baseline when the data on mushrooms and algae consumption were first measured. Among the 16,563 participants aged 65 years and above, we excluded 13 participants who did not provide information on mushrooms and algae intake and 3394 participants who did not complete at least one follow-up after the 2008 cycle. The final analyses included 13,156 eligible participants. 

### 2.2. Assessment of Mushrooms and Algae Consumption

Dietary intake was measured through a simplified food frequency questionnaire (FFQ) by face-to-face interview. The simplified FFQ in CLHLS has been recognized and used in previous studies [22,23]. Participants were asked how often they had consumed mushrooms and algae over the previous year and at age 60, respectively. The intake frequencies in CLHLS were recorded as “almost every day”, “not every day, but at least once per week”, “not every week, but at least once per month”, “not every month, but occasionally”, or “rarely or never”. We combined the categories of “almost every day” and “not every day, but at least once per week” as the “regular (≥1 time/week)” category to preserve numbers in the top category of consumption. The other two in-between groups were merged into the “occasional (>1 time/year-<4 time/month)” group. Therefore, intake frequencies of mushrooms and algae were categorized as “regular”, “occasional”, and “rare” consumption groups in the current analyses. Based on previous studies on temporal patterns of dietary intake [22,24], we further defined 9 patterns of mushrooms and algae intake from the age of 60 to the study baseline to capture the long-term temporal patterns as follows: regular-to-regular, regular-to-occasional, regular-to-rare, occasional-to-regular, occasional-to-occasional, occasional-to-rare, rare-to-regular, rare-to-occasional, and rare-to-rare. 

### 2.3. Ascertainment of Death

Our primary endpoint was all-cause mortality during the follow-up period. Information on death status and indicators of the predeath status were collected via interviews with a close family member of the deceased. The specific date of death was obtained by linkage to the official death certificate or questionnaire responses by close relatives. 

### 2.4. Assessment of Covariates

Demographic characteristics included age (years), gender (male/female), ethnicity (Han/non-Han), education (years), type of residence (urban/rural), marital status (married/not married/bereaved), and income level (in tertiles). The simplified FFQ was also used to collect dietary data on other major food groups, including fresh fruits, fresh vegetables, meat and poultry, fish and aquatic products, preserved vegetables, nuts, and legumes, as well as sugar and sweets. The Spearman correlation coefficient between intake frequency of mushrooms and algae and the abovementioned eight food groups ranged from 0.09 to 0.35 (Table S1). Other lifestyle factors included smoking status (never smoker/former smoker/current smoker), alcohol consumption (never drinker/former drinker/current drinker), and regular exercise (yes/no). Dentition status was assessed by asking about the number of natural teeth. Participants who had less than 20 remaining natural teeth were considered poor dentition, otherwise moderate-to-good dentition [25]. Health conditions were assessed by self-reported history of hypertension, diabetes, heart disease, stroke or cerebrovascular disease, cancer, and dementia. The body mass index (BMI) was calculated using measured weight and height by trained medical staff. 

### 2.5. Statistical Methods

Baseline characteristics differences of the study population were compared across mushrooms and algae consumption groups using chi-squared tests for categorical variables and analysis of variance (ANOVA) for continuous variables. Hazard ratios (HRs) and 95% confidence intervals (95% CIs) for mortality associated with mushrooms and algae consumption were estimated with the use of time-dependent Cox proportional hazards models. The follow-up period was calculated as the time from baseline assessment until the first event of death, or end of the analysis (2018). 

First, we evaluated the associations of intake frequency of mushrooms and algae with all-cause mortality, in which we continuously updated dietary information on mushrooms and algae intake. Multivariate models were adjusted for age and gender in model 1; and additionally adjusted for ethnicity, level of education, type of residence, marital status, income level, smoking status, alcohol consumption, regular exercise, and BMI in model 2; and additionally adjusted for other major food groups consumption (including fresh fruits, fresh vegetables, meat and poultry, fish and aquatic products, preserved vegetables, nuts, legumes, and sugar and sweets) in model 3. Second, to capture the role of intake behavioral changes, we determined the relative risk estimates for death associated with 9 mushrooms and algae intake patterns from the age of 60 to the study baseline. 

We performed stratified analyses by age (≤80 versus >80 years), gender (male versus female), residence (rural versus urban), smoking status (never versus former and current smokers), body mass index (<24 versus ≥24 kg/m^2^), health status (with versus without history of chronic diseases), and the number of natural teeth (<20 versus ≥20). The interactions were tested by including the interaction terms in the multivariate models. Additionally, a series of sensitivity analyses were conducted to test the robustness of the results. To control potential influence of health conditions, we further adjusted for history of major chronic diseases (e.g., hypertension, diabetes, heart disease, stroke or cerebrovascular disease, cancer, and dementia). Because major chronic diseases status may change dietary behavior or may be associated with an elevated risk of death, we also repeated the primary analyses after excluding participants with chronic diseases. In addition, to reduce potential selection bias, we instead treated participants who were lost to follow-up as censored observations at the first follow-up interval. Analyses were performed with the SAS software (version 9.4, SAS Institute). All *p*-values were two-sided and the statistical significance threshold was less than 0.05. 

## 3. Results

### 3.1. Baseline Characteristics of the Study Populations

Among 13,156 participants, the mean age was 86.9 ± 11.4 years at baseline, and males accounted for 42.6%. In total, the distribution of intake frequency of mushrooms and algae at baseline was 6291 (47.8%) for rare consumption group, 5413 (41.1%) for occasional consumption, and 1452 (11.0%) for regular consumption. Participants with higher intake frequency of mushrooms and algae were more likely to be younger, be male, be better educated, be married, live in urban, have higher income, exercise more regularly, and have a history of major chronic diseases (Table 1). In addition, in the analysis of the intake pattern from the age of 60 to the study baseline, the rare-to-rare intake pattern had the largest number of participants (*n* = 5203, 39.67%) and the regular-to-rare intake pattern had the least (*n* = 85, 0.65%).

### 3.2. Mushrooms and Algae Consumption Frequency and Mortality

During an average of 5.7 years of follow-up (74,976 person-years in total), we documented a total of 8937 death cases. After multivariate adjustment for potential confounders, an inverse association between higher intake frequency of mushrooms and algae and all-cause mortality was observed, as compared with rare consumption group (Table 2). Hazard ratios for death were 0.86 (95% CI, 0.80, 0.93) for participants who consumed mushrooms and algae at least once per week and 0.93 (95% CI, 0.89, 0.98) for those with occasional consumption (*p* for trend = 0.0001). 

### 3.3. Mushrooms and Algae Intake Patterns from Age 60 to the Study Baseline and Mortality

**Figure 1:**
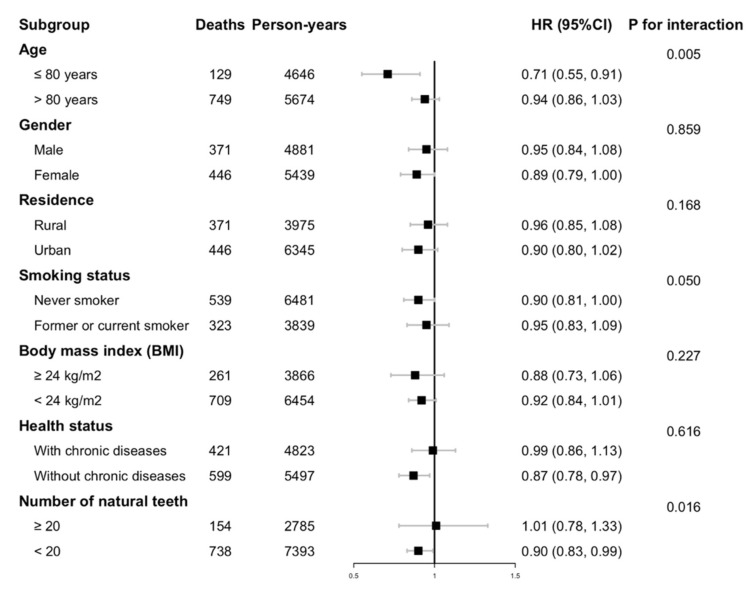
Hazard Ratios (95% CI) of regular intake of mushrooms and algae for mortality, stratified by selected characteristics. Multivariable models were adjusted for age, gender, ethnicity, level of education, type of residence, marital status, income level, smoking status, alcohol consumption, regular exercise, BMI, and major food group consumption (including fresh fruits, fresh vegetables, meat and poultry, fish and aquatic products, preserved vegetables, nuts, legumes, and sugar and sweets). Abbreviation: HR, Hazard Ratio; CI, Confidence Interval; kg, kilogram; m, meter.

As compared with participants who had rare intake patterns at both age 60 and the study baseline (average age of 86.9 years), participants who maintained regular intake pattern over time appeared to be inversely associated with all-cause mortality (HR = 0.86, 95% CI, 0.76, 0.98) (Figure 1 and Table S2). However, those in occasional-to-rare and regular-to-occasional groups had an increased risk of mortality with HRs (95% CI) of 1.18 (1.09, 1.28) and 1.15 (0.95, 1.40), respectively. In addition, we observed no significant associations between mushrooms and algae consumption at age 60 and mortality (HR = 0.96, 95% CI, 0.86, 1.07 for regular consumption compared to rare consumption), as shown in Table S3. 

### 3.4. Stratified Analyses and Sensitivity Analyses

**Figure 2:**
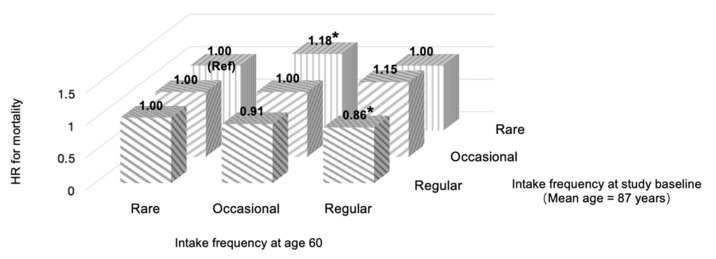
The associations with all-cause mortality of intake frequencies of mushrooms and algae at age 60 and study baseline. Multivariable models were adjusted for age, gender, ethnicity, level of education, type of residence, marital status, income level, smoking status, alcohol consumption, regular exercise, BMI, and major food group consumption (including fresh fruits, fresh vegetables, meat and poultry, fish and aquatic products, preserved vegetables, nuts, legumes, and sugar and sweets). Abbreviation: HR, Hazard Ratio; Ref, reference. * *p* value < 0.05.

In stratified analyses, the inverse associations between mushrooms and algae consumption and all-cause mortality generally persisted across subgroups according to gender, type of residence, BMI, and history of major chronic diseases (Figure 2). On the other hand, the significant associations were only observed in individuals aged 80 years and above but not in those younger than 80 years old (*p* for interaction = 0.005). A marginally significant interaction (*p* for interaction = 0.050) was observed for smoking status, with associations that appeared to be null in those who were former or current smokers. We also noted significant interaction between dentition status and mushrooms and algae consumption with respect to the risk of death; an inverse association was observed for participants who had poor dentition (less than 20 natural teeth) but not for those who had moderate-to-good dentition (20 natural teeth or more) (*p* for interaction = 0.016).

The results remained generally unchanged compared with the primary results when we repeated the analyses: (1) with additional adjustment for history of chronic diseases at baseline; (2) excluding participants with history of chronic diseases at baseline; and (3) treating participants who were lost to follow-up as censored observations at the first follow-up interval (Table S4). 

## 4. Discussion

In this large prospective cohort study of Chinese older adults, we observed an inverse association between long-term consumption of mushrooms and algae and total mortality, after adjustment for sociodemographic, lifestyle, and other dietary factors. As compared with participants who rarely consumed mushrooms and algae, those who consumed regularly (at least once per week) had a 14% lower risk of death. Inverse associations persisted among major subgroups across gender, type of residence, BMI, and history of major chronic diseases. Furthermore, we observed that participants who maintained regular consumptions of mushrooms and algae both at age 60 and the study baseline had the lowest risk of total mortality. 

Our results are generally consistent with those of a recent meta-analysis showing that higher consumption of mushrooms was associated with a 6% lower risk of all-cause mortality [16]. Specifically, among five studies included in the meta-analyses, two large-scale studies of western populations reported statistically significant associations. Specifically, in the Third National Health and Nutrition Examination Survey (NHANES III), the hazard ratio for total mortality among a nationally representative sample of US adults who consumed mushrooms was 0.84 (95% CI, 0.73, 0.98), as compared to those with no consumption [17]. Similarly, in the European Prospective Investigation Into Cancer and Nutrition (EPIC) study, the corresponding HR of death for participants with the highest tertile of mushrooms consumption was 0.94 (95% CI, 0.90, 0.98) than those at the lowest tertile [18]. However, the other three studies yielded nonsignificant results [16,19,20]; one study was from NHANES (2003–2014) and two studies were from Japan. The discrepancy may be influenced by different sample sizes (ranged from 799 to 451,151), different assessment methods of mushrooms and algae intake, inadequate control for confounding, and different dietary cultures. The present study, conducted among 13,156 Chinese older adults, found a 7–15% lower risk of all-cause mortality in those who had more frequent consumption of mushrooms and algae. Our findings provide additional evidence to the potential benefit of mushrooms and algae consumption for healthy longevity among one of the largest Asian populations. Nevertheless, further studies are warranted to identify the optimal level and specific types of mushrooms and algae consumption in a healthy diet. 

We also noted that participants who maintained a regular-to-regular intake pattern of mushrooms and algae from age 60 to the study baseline (average age of 87 years old) had the lowest risk of death compared to those with a rare-to-rare intake pattern. In contrast, individuals with a decreased intake pattern from occasional-to-rare consumption had an 18% higher risk of mortality. These results underscore the importance of maintaining a stable and regular intake pattern of mushrooms and algae over time. This also has important public health implications, as the intake of mushrooms and algae at least once a week may be feasible as an integral part of a healthy diet. 

Several biological mechanisms may explain the observed association between mushrooms and algae consumption and all-cause mortality. Emerging evidence demonstrated the health benefits of antioxidant and anti-inflammation properties of mushrooms and algae. For example, previous researchers have highlighted the bioavailability of ergothioneine, as a natural antioxidant on human health [6,26,27]. A retrospective study has demonstrated that consuming 100 g of white button mushrooms per day for 16 weeks was associated with higher serum ergothioneine concentrations along with higher antioxidant markers of ORAC (oxygen radical absorption capacity) and adiponectin, as well as lower circulating oxidative stress factors [26]. Recent studies have demonstrated the physiological activities of polysaccharides from mushrooms and algae including immunomodulatory action, antioxidant activity, anti-inflammatory actions, and anticarcinogenic [8,28]. Moreover, algae also represents one of the richest sources of natural antioxidants among marine resources [29,30]. Given the interrelation of oxidative stress and inflammation with the initiation and progression of several chronic diseases [31], mushrooms and algae intake may reduce the risk of mortality by influence the incidence of common chronic diseases, such as cardiovascular disease, cancers, degenerative disease, and metabolic syndrome.

Strengths of our study include the prospective study design and the use of a large, nationally representative sample. However, this study has certain limitations. First, frequency of mushrooms and algae consumption was estimated using a non-quantitative FFQ without detailed information on the intake amount. For the same reason, we could not control for total energy in the multivariable models, and residual confounding could not be completely ruled out. Second, the intake frequency of mushrooms and algae at age 60 were recalled at the study baseline, therefore some measurement error is inevitable. Third, there is no information on the intake frequency of the specific types of mushrooms and algae in the CLHLS study. According to the Dietary Guideline for Chinese residents (2022), the commonly consumed types of mushrooms and algae include shiitake mushroom, button mushroom, oyster mushroom, *Auricularia auricula-judae* (commonly called black woody ear), *Tremella fuciformis*, kelps, and *Undaria pinnatifida*. Further studies with more detailed dietary assessments are needed to elucidate the associations. Finally, restriction of the study sample to Chinese older adults may limit the generalizability to other populations.

In conclusion, our study findings support the potential beneficial roles of mushrooms and algae in reducing all-cause mortality among Chinese older adults. Maintaining a stable pattern with regular consumption of mushrooms and algae during mid–to-late life demonstrated the strongest benefit against death. Further studies are warranted to evaluate the health benefits for longevity of specific types of mushrooms and algae.

## Figures and Tables

**Table 1:** Baseline characteristics of the study participants.

Variables	Total	Rare (0–1 Time/Year)	Occasional (>1 Time/Year-<4 Time/Month)	Regular (≥1 Time/Week)	*p*-Value
No.	13,156	6291	5413	1452	
Age (years), mean (SD)	86.9 (11.4)	88.0 (11. 0)	86.1 (11.4)	85.2 (12.1)	<0.001
Gender, *n* (%)					0.010
Male	5605 (42.6)	2607 (41.4)	2337 (43.2)	661 (45.5)	
Female	7551 (57.4)	3684 (58.6)	3076 (56.8)	791 (54.5)	
Ethnic groups, *n* (%)					<0.001
Han	12,282 (93.4)	5790 (92.0)	5080 (93.8)	1412 (97.2)	
Non-Han	874 (6.6)	501 (8.0)	333 (6.2)	40 (2.8)	
School (years), mean (SD)	1.95 (3.3)	1.51 (2.8)	2.06 (3.4)	3.43 (4.6)	<0.001
Residential area, *n* (%)					<0.001
Urban	4812 (36.6)	1848 (29.4)	2090 (38.6)	874 (60.2)	
Rural	8344 (63.4)	4443 (70.6)	3323 (61.4)	578 (39.8)	
Marital status, *n* (%)					<0.001
Married	4393 (33.4)	1949 (31.0)	1893 (35.0)	551 (37.9)	
Not married	144 (1.1)	85 (1.4)	44 (0.8)	15 (1.0)	
Bereaved	8619 (65.5)	4257 (67.7)	3476 (64.2)	886 (61.0)	
Annual household income, *n* (%)					<0.001
Low (<6000 yuan)	4243 (32.3)	2579 (41.1)	1468 (27.2)	196 (13.5)	
Medium (≥6000 and <20,000 yuan)	4070 (31.0)	1858 (29.6)	1837 (34.0)	375 (25.9)	
High (≥20,000 yuan)	4825 (36.7)	1845 (29.4)	2101 (38.9)	879 (60.6)	
Smoking status, *n* (%)					<0.001
Never	8714 (66.2)	4276 (68.0)	3530 (65.2)	908 (62.5)	
Former	2065 (15.7)	920 (14.6)	866 (16.0)	279 (19.2)	
Current	2377 (18.1)	1095 (17.4)	1017 (18.8)	265 (18.3)	
Alcohol consumption, *n* (%)					<0.001
Never	9009 (68.5)	4449 (70.7)	3603 (66.6)	957 (65.9)	
Former	1787 (13.6)	803 (12.8)	758 (14.0)	226 (15.6)	
Current	2360 (17.9)	1039 (16.5)	1052 (19.4)	269 (18.5)	
Regular exercise, *n* (%)	3656 (27.8)	1467 (23.3)	1518 (28.0)	671 (46.2)	<0.001
BMI (kg/m^2^), mean (SD)	20.6 (16.8)	20.1 (16.7)	20.9 (16. 8)	21.6 (17.2)	0.002
Hypertension, *n* (%)	2518 (19.1)	1125 (17.9)	1032 (19.1)	361 (24.9)	<0.001
Diabetes, *n* (%)	296 (2.2)	94 (1.5)	138 (2.5)	64 (4.4)	<0.001
Heart disease, *n* (%)	1118 (8.5)	417 (6.6)	469 (8.7)	232 (16.0)	<0.001
Stroke, *n* (%)	749 (5.7)	327 (5.2)	299 (5.5)	123 (8.5)	<0.001
Cancer, *n* (%)	42 (0.3)	14 (0.2)	13 (0.2)	15 (1.0)	<0.001
Dementia, *n* (%)	251 (1.9)	139 (2.2)	91 (1.7)	21 (1.4)	0.045

Abbreviation: SD, standard deviation; BMI, body mass index; kg, kilogram; m, meter.

**Table 2:** Hazard Ratios (95% CI) for mortality according to baseline intake frequency of mushrooms and algae.

Models	Rare (0–1 Time/Year)	Occasional (>1 Time/Year-<4 Time/Month)	Regular (≥1 Time/Week)	*p*-Trend
Deaths	4546	3574	817	-
Person-years	32,191	29,019	10,320	-
Model 1 ^a^	1 (Ref)	0.91 (0.87, 0.95)	0.80 (0.74, 0.85)	<0.0001
Model 2 ^b^	1 (Ref)	0.91 (0.87, 0.96)	0.84 (0.78, 0.90)	<0.0001
Model 3 ^c^	1 (Ref)	0.93 (0.89, 0.98)	0.86 (0.80, 0.93)	0.0001

^a^ Model 1: adjusted for age and gender. ^b^ Model 2: additionally adjusted for ethnicity, level of education, type of residence, marital status, income level, smoking status, alcohol consumption, regular exercise, and BMI. ^c^ Model 3: additionally adjusted for dietary confounding variables, including fresh fruits, fresh vegetables, meat and poultry, fish and aquatic products, preserved vegetables, nuts, legumes, and sugar and sweets. Abbreviation: CI, confidence interval.

## Data Availability

Data described in this paper are stored in the Peking University Open Research Data Platform, a public data repository (https://opendata.pku.edu.cn/dataset.xhtml?persistentId=doi:10.18170/DVN/WBO7LK; accessed on 21 November 2020).

## Supplementary Materials

The following supporting information can be downloaded at: https://www.mdpi.com/article/10.3390/nu14193891/s1, Table S1: Correlation coefficient between mushrooms and algae and other food groups, Table S2: Hazard Ratios (95% CI) for mortality according to mushrooms and algae intake patterns from age 60 to the study baseline, Table S3: Hazard Ratios (95% CI) for mortality according to intake frequency of mushrooms and algae at age 60, Table S4: Sensitivity analysis for the association of mushrooms and algae intake frequency with mortality.
