# Supplementary material for: Association of Mushrooms and Algae Consumption with Mortality among Chinese Older Adults: A Prospective Cohort Study
Source: Nutrients. 2022 Sep 20;14(19):3891. doi: 10.3390/nu14193891 (PMC9571415; doi:10.3390/nu14193891)
Supplement: Supplementary file 1 [file nutrients-14-03891-s001.zip › nutrients-1886899-supplementary.pdf]

Supplementary Materials

Table S1. Correlation coefficient between mushrooms and algae and other food groups.

Food groups	Mushrooms and Algae
Fresh fruits	0.24
Fresh vegetables	0.15
Meat	0.13
Fish	0.21
Nut	0.35
Legumes	0.22
Preserved vegetables	0.09
Sugar and sweets	0.12

Table S2. Hazard Ratios (95% CI) for mortality according to mushrooms and algae intake patterns from age 60 to the study baseline.

Intake frequency patterns	Death	Person-years	HR (95% CI)
Rare-Rare	3704	28532	Ref
Rare-Occasional	1072	7606	1.00 (0.93, 1.08)
Rare-Regular	230	1562	1.00 (0.87, 1.16)
Occasional-Rare	766	4632	1.18 (1.09, 1.28)
Occasional-Occasional	2371	23116	1.00 (0.95, 1.06)
Occasional-Regular	247	2547	0.91 (0.79, 1.05)
Regular-Rare	57	465	1.00 (0.77, 1.31)
Regular-Occasional	123	1062	1.15 (0.95, 1.40)
Regular-Regular	336	5161	0.86 (0.76, 0.98)

Multivariable models were adjusted for age, gender, ethnicity, level of education, type of residence, marital status, income level, smoking status, alcohol consumption, regular exercise, BMI, mortality-related dietary variables (including fresh fruits, fresh vegetables, meat and poultry, fish and aquatic products, preserved vegetables, nuts, legumes, and sugar and sweets). Abbreviation: HR, Hazard Ratio; CI, Confidence Interval.

Table S3. Hazard Ratios (95% CI) for mortality according to intake frequency of mushrooms and algae at age 60.

Models	Rare (0-1 time per year)	Occasional (>1 time/year-<4 time/month)	Regular (≥ 1 time/week)	P-trend
Deaths	5006	3384	516	-
Person-years	37700	30295	6688	-
Model 1	1 (Referent)	0.99 (0.95, 1.03)	0.81 (0.74, 0.89)	0.022
Model 2	1 (Referent)	1.02 (0.97, 1.07)	0.93 (0.84, 1.02)	0.695
Model 3	1 (Referent)	0.99 (0.94, 1.04)	0.96 (0.86, 1.07)	0.488

Model 1: adjusted for age, gender. Model 2: additionally adjusted for ethnicity, level of education, type of residence, marital status, income level, smoking status, alcohol consumption, regular exercise, and BMI. Model 3: additionally adjusted for major food group consumption, including meat and poultry, fish and aquatic products, preserved vegetables, nuts, legumes, and sugar and sweets at age 60. Abbreviation: CI, confidence interval.

Table S4. Sensitivity analyses for the association of mushrooms and algae intake frequency with mortality.

Intake frequency	Deaths	Person-years	HR (95% CI)	P-value
**Sensitivity analysis 1*: Further adjusted for chronic diseases, including hypertension, diabetes, heart disease, stroke or cerebrovascular disease, cancer, and dementia.**				
Rare	4546	33735	Ref	-
Occasional	3574	31828	0.98 (0.93, 1.03)	0.387
Regular	817	9294	0.88 (0.81, 0.96)	0.003
**Sensitivity analysis 2: Excluding participants with chronic diseases.**				
Rare	3328	24482	Ref	-
Occasional	2557	22203	0.98 (0.93, 1.04)	0.477
Regular	537	5618	0.88 (0.79, 0.98)	0.017
**Sensitivity analysis 3: treating participants who were lost to follow-up as censored observations at the first follow-up interval.**				
Rare	1917	28793	Ref	-
Occasional	1637	28187	0.99 (0.92, 1.07)	0.754
Regular	364	8449	0.87 (0.76, 0.99)	0.034

Multivariable models were adjusted for age, gender, ethnicity, level of education, type of residence, marital status, and income level, smoking status, alcohol consumption, regular exercise, BMI, and major food group consumption (including fresh fruits, fresh vegetables, meat and poultry, fish and aquatic products, preserved vegetables, nuts, legumes, and sugar and sweets).

* Further adjusted for chronic diseases, including hypertension, diabetes, heart disease, stroke or cerebrovascular disease, cancer, and dementia.

Abbreviation: HR, Hazard Ratio; CI, Confidence Interval.
